# Reliability and validity of a novel quality of life questionnaire for female patients with adolescent idiopathic scoliosis: Scoliosis Japanese Questionnaire-27: a multicenter, cross-sectional study

**DOI:** 10.1186/s12891-018-2025-7

**Published:** 2018-04-03

**Authors:** Toru Doi, Hirokazu Inoue, Yasuhisa Arai, Osamu Shirado, Tokuhide Doi, Ken Yamazaki, Koki Uno, Haruhisa Yanagida, Katsushi Takeshita

**Affiliations:** 1 Department of Orthopaedic Surgery, University of Tokyo, 7-3-1 Hongo, Bunkyo-ku, Tokyo, Japan; 2 Department of Orthopaedic Surgery, Jichi Medical University, 3311-1 Yakushiji, Shimotsuke, Tochigi 329-0498 Japan; 3 Tokyo Metropolitan Rehabilitation Hospital, 2-14-1 Tsutsumidori, Sumida-ku, Tokyo, Japan; 4 Department of Orthopaedic and Spinal Surgery, Aizu Medical Center, Fukushima Medical University, 21-2 Kawahigashimachitanisawa, Aizuwakamatsu, Fukushima Japan; 5 Shizu Clinic, 1669 Kamishizu, Sakura, Chiba Japan; 6 Iwate Spinal Scoliosis Center, 103-1 Ogamayoshimizu, Takizawa, Iwate Japan; 7 Department of Orthopaedic Surgery, National Hospital Organization Kobe Medical Center, 3-1-1 Nishiochiai, Suma-ku, Kobe, Hyogo Japan; 8 Fukuoka Children’s Hospital, 5-1-1 Kashiiteriha, Higashi-ku, Fukuoka, Japan

**Keywords:** Adolescent idiopathic scoliosis, Questionnaires, Patient-reported outcome measure, Validation study, Reliability study, Scoliosis Japanese Questionnaire-27, Scoliosis Research Society-22, Health-related quality of life, AIC network

## Abstract

**Background:**

A progressive deformity associated with adolescent idiopathic scoliosis (AIS) negatively affects a patient’s health-related quality of life (HRQOL). Although the Scoliosis Research Society-22 (SRS-22) is the standard measurement tool for assessing HRQOL in patients with AIS, it is partially suboptimal for evaluating HRQOL in Japanese patients with AIS because of cultural differences. The purpose of this study was to develop a novel patient-reported outcome measure for Japanese female patients with AIS and to evaluate the reliability and validity of this questionnaire in comparison with the SRS-22 tool.

**Methods:**

We developed 27 questions based on the psychosocial problems in the daily life of young female patients with AIS in Japan, the Scoliosis Japanese Questionnaire-27 (SJ-27). To evaluate its reliability, the internal consistency was assessed using Cronbach’s alpha coefficient. Concurrent validity was evaluated using Spearman’s correlation coefficient between the SJ-27 and the SRS-22. To investigate the construct validity of the SJ-27, the correlation between the SJ-27 questions was assessed using Akaike’s information criterion (AIC).

**Results:**

We analyzed 384 female patients with AIS. Cronbach’s alpha coefficients were 0.914 and 0.829 for the SJ-27 and the SRS-22, respectively. Spearman’s correlation coefficient between the SJ-27 and the SRS-22 was 0.692 (*p* < 0.001). The AIC analysis indicated that the SJ-27 items are divided into five domains, indicating that the SJ-27 covered a wide range of health-related problems among female patients with AIS.

**Conclusions:**

The results suggest that the SJ-27 is a reliable and valid patient-reported outcome measure for evaluating HRQOL in female patients with AIS in Japan.

## Background

Adolescent idiopathic scoliosis (AIS) is the most common childhood-onset spinal deformity and is present in 2–3% of adolescents worldwide [1]. A curve progression of >30^o^ is more commonly observed in female patients than in male patients [2]. A recent study of AIS prevalence in Japan showed that the overall prevalence in school-age children (11–14 years) is 0.87% [3].

The progressive spinal deformity associated with AIS has been reported to negatively affect a patient’s health-related quality of life (HRQOL) [4–6], with increased back pain, reduced physical activity, and psychosocial problems being associated with a disfigured appearance [7–10].

Patient-reported outcome measures are useful instruments for assessing patients’ HRQOL [11] and are widely used for patients with orthopedic diseases [12–14], including spinal disorders [15]. The Scoliosis Research Society (SRS) patient-reported outcome measure has been established as a practical and disease-specific measure for assessing HRQOL in patients with AIS [16–19]. An earlier version of the SRS Questionnaire, SRS-24, was developed by Haher et al. [20]. The latest version, SRS-22, which was developed by Asher in 2003 [18], is a practical questionnaire for facilitating the assessment of HRQOL in patients with AIS and has been reported to have a high reliability and validity [16–19, 21]. The SRS-22 questionnaire was revised in 2006 (SRS-22r) [22] in order to improve the internal consistency in the Function domain and this revised version of the SRS-22 questionnaire is commonly used worldwide for the assessment of HRQOL in patients with AIS. The SRS-22 questionnaire covers four domains (Function, Pain, Self image, Mental health) consisting five questions each and one domain (Satisfaction/Dissatisfaction with management) consisting two questions. Each question has five verbal response alternatives ranging from 1 (worst) to 5 (best). The results of the SRS-22 questionnaire are expressed as the mean (total sum of the domain divided by the number of items answered) for each domain and for the total score (minimum: 1 point, maximum: 5 points). To apply the SRS-22 questionnaire in non-English speaking countries, translated and adapted versions of the SRS-22 questionnaire have been developed, and their reliability and validity have been reported [23–34]. For Japanese patients with AIS, the SRS-24 was translated into Japanese; however, previous studies demonstrated that its internal consistency and discriminative validity are not optimal [35, 36], showing definite differences between American and Japanese patients in all the domain scores. These differences may reflect the cultural differences between the assessed populations [37]. The Japanese version of the SRS-22 questionnaire was established in 2007, and its reliability and validity were approved [38]. However, in comparison to its corresponding scale in the original English version, the self-image subscale showed a different pattern of correlation with other variables. This resulted in the study authors suggesting further psychometric assessments to better understand clinical HRQOL. Basically, the self-image subscale questions in the SRS-22 (e.g., Q19 “Do you feel attractive with your current back condition?”) are thought to be suboptimal for the modest or reserved personality types observed in a majority of Japanese people. Moreover, the question related to economic burden (Q15 “Are you and/or your family experiencing financial difficulties because of your back?”) is also suboptimal for Japanese patients due to the different types of medical insurance systems in Japan, relative to the USA. A previous study on the Turkish version of the SRS-22 reported that Q15 had poor internal consistency, resulting in this item being omitted from this version of the questionnaire [23]. Thus, we sought to develop a novel, patient-reported outcome measure for patients with AIS that is adapted to the Japanese population. The purpose of this study was to develop and introduce a new and unique patient-reported outcome measure for assessing HRQOL in Japanese female patients with AIS and to evaluate its validity and reliability.

## Methods

### Development of the Scoliosis Japanese Questionnaire-27 (SJ-27)

An original patient-reported outcome measure for Japanese female patients with AIS, the SJ-27, was developed and refined over 17 sessions by an expert committee of the Japanese Scoliosis Society consisting 10 specialists in spinal surgery, rehabilitation, pediatric psychiatrics, and biostatistics. A group of three Japanese Scoliosis Society board-certified experts with extensive spinal disorder treatment experience repeatedly discussed and selected items based on their relevance to the aims of the questionnaire. These experts also sought the advice of a professional pediatric psychiatrist regarding items related to psychosocial problems among adolescent patients with AIS. During the development of the questionnaire, the focus was directed to young female patients because the curve progression and prevalence are greater in females than in males. Additionally, a progressive spinal deformity can result in greater appearance-related psychological problems in females than in males [7, 39]. Furthermore, we also focused on the discomfort when wearing clothes or underwear caused by the spinal deformity because this is an important problem in the daily life of young female patients. To develop the items related to such problems, we interviewed a few young Japanese female patients with AIS and took their opinions into consideration for the development of the SJ-27. Ultimately, each SJ-27 question essentially reflects the lifestyle of young female patients in Japan.

### SJ-27 contents

SJ-27 is a self-administered, disease-specific outcome measure that consists of 27 items (Table 1), summarized as follows:

1. Four questions regarding back pain while lying down and after sitting or standing, or neck/shoulder stiffness or soreness (Q1–4)
2. Seven questions regarding discomfort while wearing clothes or holding bags (Q5–10 and 27)
3. Four questions regarding difficulties associated with participating in physical activities (Q11, 15, 16, and 19)
4. Six questions regarding being self-conscious about one’s appearance while in public (Q12–14, 22, 25, and 26)
5. Six questions related to feelings of anxiety or depression due to the spinal deformity (Q17, 18, 20, 21, 23, and 24)

**Table 1:** Contents of the Scoliosis Japanese Questionnaire-27

Please fill out the following questions about your upper and lower back from the past month. Tick the most appropriate answer for each question. If you wear a brace, answer the questions for the times when you were not wearing the brace.
1. To what extent do you feel pain in your upper or lower back while lying down? □Not at all □Mild □Moderate □Considerable □Severe
2. To what extent do you feel pain in your upper or lower back after sitting for an hour? □Not at all □Mild □Moderate □Considerable □Severe
3. To what extent do you feel pain in your upper or lower back after standing for long periods? □Not at all □Mild □Moderate □Considerable □Severe
4. To what extent do you feel stiffness or soreness in your shoulders or neck? □Not at all □Mild □Moderate □Considerable □Severe
5. To what extent do you feel uncomfortable when wearing a jacket or coat? □Not at all □Mild □Moderate □Considerable □Severe
6. To what extent are you concerned about the waistline of a skirt? □Not at all □Mild □Moderate □Considerable □Severe
7. To what extent do you have difficulty putting on socks or stockings? □Not at all □Mild □Moderate □Considerable □Severe
8. To what extent are you concerned about the fit of T-shirts? □Not at all □Mild □Moderate □Considerable □Severe
9. To what extent are you bothered by the slipping of bra or camisole straps from your shoulders? □Not at all □Mild □Moderate □Considerable □Severe
10. To what extent are you self-conscious about the curve of your back when wearing lighter clothing in warmer weather? □Not at all □Slightly □Moderately □Strongly □Extremely
11. Do you find yourself wanting to avoid exercise? □Not at all □Slightly □Moderately □Strongly □Extremely
12. Have parents, friends, or teachers told you that you have a back problem? □Never □Rarely □Sometimes □Often □Very often
13. Do you feel embarrassed to bathe with your mother or sisters because of the curve of your back? □Not at all □Slightly □Somewhat □Quite □Very much
14. How self-conscious are you about classmates or colleagues noticing your appearance? □Not at all □Slightly □Somewhat □Quite □Very much
15. Do you become anxious when standing in front of a group of people? □Not at all □Slightly □Somewhat □Quite □Very much
16. To what extent are you reluctant to participate in sporting events or performances? □Not at all □Slightly □Somewhat □Quite □Very much
17. To what extent does your appearance in the mirror make you self-conscious? □Not at all □Slightly □Somewhat □Quite □Very much
18. To what extent are you self-conscious about the x-ray images on your back? □Not at all □Slightly □Somewhat □Quite □Very much
19. Do you feel sports or exercise will have a negative effect on your spine? □Not at all □Slightly □Somewhat □Quite □Very much
20. Do you worry about your future regarding marriage, childbirth, and your children inheriting your condition? □Not at all □Slightly □Somewhat □Quite □Very much
21. To what extent do you wish you could change the current condition of your spine? □Not at all □Slightly □Somewhat □Quite □Very much
22. To what extent do you feel shy or reclusive because of the shape of your spine? □Not at all □Slightly □Somewhat □Quite □Very much
23. To what extent do you feel depressed due to the condition of your spine? □Not at all □Slightly □Somewhat □Quite □Very much
24. To what extent do you feel an inferiority complex due to the shape of your spine? □Not at all □Slightly □Somewhat □Quite □Very much
25. To what extent do you feel overly self-conscious about your appearance due to the shape of your spine? □Not at all □Slightly □Somewhat □Quite □Very much
26. To what extent do you think of yourself as less capable than others because of your spine? □Not at all □Slightly □Somewhat □Quite □Very much
27. To what extent do you feel self-conscious about how you hold bags? □Not at all □Slightly □Somewhat □Quite □Very much

The SJ-27 is intended to cover a wide range of HRQOL issues among female patients with AIS. The 27 items are scored on a 5-point scale from no impairment (0 point) to severe impairment (4 points) and then added to yield a total score (minimum: 0 point, maximum: 108 points). We adopted the scoring method whereby a higher score indicates a much worse HRQOL, which is opposite to the scoring method of the SRS-22. The reason why we adopted the opposite scoring method to the SRS-22 is because we hope to adopt the same scoring method as the visual analog scale (VAS) or Oswestry disability index (ODI), which are broadly utilized patient-reported outcome instruments in spine disease.

### Study design and patient recruitment

This study is a multicenter cross-sectional analysis designed to assess the reliability and validity of the SJ-27. Female participants with AIS were recruited from outpatient clinics at 24 institutions located throughout Japan between July 2009 and June 2011. These patients were invited to participate in this study regardless of severity and variation in treatment methods. The inclusion criteria were as follows:

1. Young women 10 to 18 years of age.
2. Radiographic findings of a main curve Cobb angle >10^o^.
3. Diagnosis of AIS confirmed by experienced specialists.

Patients who were unable to respond to the Japanese questionnaire were excluded. The adequate sample size was calculated using the following simple formula [40]: *n* = *Z*^2^ *P*(1-*P*)/*d*^2^ where *n* is the sample size, *Z* is the statistic corresponding to the level of confidence (for the 95% level of confidence, which is conventional, the *Z* value is 1.96), *P* is the expected response rate (a *P* value of 50% was applied because it would result in a larger sample size), and *d* is the precision (a precision of 5% was applied if a *P* value is between 10% and 90%). Accordingly, the sample size computed using the given formula resulted in 384 participants. We thus aimed to enroll approximately 400 participants in this study.

### Testing the questionnaire

Patients invited to participate in this study were given SJ-27 questionnaire booklets and copies of the Japanese version of the SRS-22. It takes approximately 5 min to complete both questionnaires. In the questionnaire instructions, we specified that patients must complete both questionnaires by themselves, but provided that if there were a need for help in filling out the questionnaire, their parents or attendants may help them fill out the questionnaire, but the answer must be patient’s own. The attending physicians recorded the patient’s AIS treatment history, including observation, therapeutic exercise, brace, surgery, and other treatments (multiple choices allowed).

### Spinal radiographic examination

Standing anteroposterior radiographs of the whole spine were taken to confirm the AIS diagnosis. The Cobb method was used to measure curve angles at the time the questionnaire was administered.

### Statistical analysis

Floor and ceiling effects for the total SJ-27 score and mean total SRS-22 score were examined by calculating the percentage of patients who achieved the lowest (SJ-27: 0 point, SRS-22: 1 point) or the highest (SJ-27: 108 points, SRS-22: 5 points) score, respectively. We adopted the commonly used 15% threshold for the percentage of patients achieving the lowest or the highest score to define floor and ceiling effects [41, 42]. Cronbach’s alpha coefficients were calculated for both scales to indicate internal consistency. A Cronbach’s alpha coefficient of 0.7 or higher was considered acceptable for internal consistency, while a score above 0.8 was good and above 0.9 was excellent [43]. Cronbach’s alpha coefficients were also used to assess how each item contributed to internal consistency by recalculating the Cronbach’s alpha coefficient upon deletion of each item in the SJ-27 respectively and then comparing this to the Cronbach’s alpha coefficient for all items. Furthermore, we evaluated Cronbach’s alpha coefficients for the top three treatment groups in both questionnaires to examine whether each treatment method for AIS affects the internal consistency.

To evaluate the concurrent validity, the correlation between the SJ-27 and the SRS-22 was assessed using Spearman’s correlation coefficient. The correlation coefficient was interpreted as follows: ±0.1 was considered weak, ±0.3 moderate, and ± 0.5 a strong correlation [43].

To evaluate the construct validity of the SJ-27, the correlation between SJ-27 questions was evaluated using categorical principal component analysis (CATPCA) and Akaike’s information criterion (AIC) [44, 45]. The AIC values were calculated for all combinations of questions and sorted in ascending order using a categorical data analysis program (CATDAP)-02 developed by the Institute of Statistical Mathematics in Japan [46]. CATDAP-02, running with R language software, simultaneously searched for the best subset and categorization of explanatory variables and automatically indicated matching combinations using AIC. SJ-27 construct structures, identified using CATPCA, and AIC factor plotting were performed using IBM SPSS Statistics, version 23 (IBM Corp, Armonk, NY, USA) and an open-source graph visualization software (Graphviz, http://www.graphviz.org), respectively.

## Results

### Patient characteristics

A total of 405 female patients diagnosed with AIS were recruited, and 21 patients over 18 years of age were excluded. Thus, 384 participants were enrolled in this study. Table 2 shows the demographic characteristics of the 384 participants and the number of participants in each institution. The mean patient age was 14.3 (standard deviation (SD), 1.9) years, and the mean Cobb angle was 31.0^o^ (SD, 12.6). The AIS treatment history survey showed that 161 patients were treated using braces, 120 were observed, and 78 underwent surgery, respectively.

**Table 2:** Demographic characteristics of the participants and the number of participants in each institution

		Patients with AIS (*n* = 384)	
Mean age (SD), years		14.3 (1.9)	
Mean body mass (SD), kg		45.0 (7.2)	
Mean body height (SD), cm		155.7 (7.1)	
Mean body mass index (SD), kg / m2		18.5 (2.3)	
Treatment, n (multiple choices allowed)		31.0 (12.6)	
Mean Cobb angle (SD), degrees		14.3 (1.9)	
Mean age (SD), years		45.0 (7.2)	
Treatment, n (multiple choices allowed)			
Observation		120	
Exercise		28	
Brace		161	
Surgery		78	
Other		23	
Institution, n			
Okayama Medical Center	10	Shiba Clinic	14
Aizu Medical Center	2	Jikei University Hospital	20
Iwate Medical University Hospital	16	Akita University Hospital	8
Gifu University Hospital	17	Juntendo University Hospital	26
Kurume University Hospital	3	Niigata Spine Surgery Center	19
University of Miyazaki Hospital	20	Niigata University Medical and Dental Hospital	9
Kanazawa Medical University Hospital	5	Gunma Spine Center of Harunaso Hospital	20
Keio University Hospital	23	Sendai Nishitaga Hospital	20
Hirosaki University Hospital	14	Tokai University Hospital	18
Hirosaki Memorial Hospital	5	University of Tokyo Hospital	26
Saitama Medical University Hospital	30	Hamamatsu University Hospital	20
Saitama Medical Center	19	Fukuoka Children’s Hospital	20

### Floor and ceiling effects

The floor and ceiling effects for the total SJ-27 score and mean total SRS-22 score were evaluated in this patient group. A total of 370 out of 384 participants (96.4%) completed the SJ-27, while a total of 367 out of 384 participants (95.6%) completed the SRS-22. The mean score in the SJ-27 and the SRS-22 were 23.2 (SD, 16.7) and 4.1 (SD, 0.4), respectively. The minimum and maximum points of both scales were 0.0 and 86.0 points, respectively, for the SJ-27 and 2.9 and 5.0 points, respectively, for the SRS-22. The percentages of patients who achieved the lowest score were 0.5% for the SJ-27 and 0.0% for the SRS-22, respectively. The percentages of patients who achieved the highest score were 0.0% for the SJ-27 and 0.3% for the SRS-22, respectively.

### Internal consistency

The response completion rates were 99.9% for the SJ-27 and 99.8% for the SRS-22, respectively. Cronbach’s alpha coefficients were 0.914 for the SJ-27 (14 of the 384 participants were excluded due to missing or incomplete answers) and 0.829 for the SRS-22 (17 of the 384 participants were excluded due to missing or incomplete answers), respectively. Table 3 shows the Cronbach’s alpha coefficient upon deletion of each item in the SJ-27. Only when Q16 was experimentally deleted, Cronbach’s alpha coefficient increased slightly to 0.915 (Table 3). Cronbach’s alpha coefficient showed a minimum value of 0.907 when Q10 or Q17 was deleted from the SJ-27 (Table 3). Cronbach’s alpha coefficients in each of the top three treatment groups (brace, observation, and surgery) were 0.909, 0.903, and 0.916, respectively, for the SJ-27 and 0.506, 0.395, and 0.606, respectively, for the SRS-22.

**Table 3:** Cronbach’s alpha coefficient upon deletion of each item in the Scoliosis Japanese Questionnaire-27

	Scale mean if item is deleted	Scale variance if item is deleted	Corrected item - total correlation	Cronbach’s alpha if item is deleted
Q01	48.76	2 6 9 .2 0 9	.409	.912
Q02	48.20	2 6 0 .0 9 7	.525	.911
Q03	48.23	2 6 1 .9 1 7	.473	.911
Q04	47.82	2 6 2 .7 3 6	.349	.914
Q05	48.57	2 5 7 .8 5 0	.644	.909
Q06	48.43	2 5 6 .3 4 3	.588	.909
Q07	48.78	2 7 0 .1 3 7	.317	.914
Q08	48.54	2 5 8 .2 2 2	.600	.909
Q09	48.30	2 5 6 .6 5 6	.611	.909
Q10	48.35	2 5 1 .9 6 7	.729	.907
Q11	48.34	2 6 5 .7 3 3	.335	.914
Q12	48.12	2 6 1 .3 7 9	.430	.912
Q13	49.03	2 7 3 .2 2 9	.325	.913
Q14	48.68	2 5 8 .5 4 2	.663	.909
Q15	47.99	2 6 1 .8 2 7	.382	.914
Q16	48.59	2 7 1 .3 9 8	.234	.915
Q17	48.23	2 5 2 .7 1 4	.694	.907
Q18	47.10	2 5 4 .5 6 0	.560	.910
Q19	48.56	2 6 5 .0 8 2	.445	.912
Q20	48.30	2 5 7 .5 8 2	.575	.910
Q21	46.70	2 6 0 .1 0 3	.396	.914
Q22	48.90	2 6 7 .5 2 3	.552	.911
Q23	48.28	2 5 3 .5 3 9	.653	.908
Q24	48.34	2 5 3 .8 5 5	.659	.908
Q25	48.50	2 5 4 .5 7 0	.689	.908
Q26	48.63	2 6 2 .2 1 2	.545	.910
Q27	48.11	2 5 9 .6 2 0	.486	.911

### Concurrent and construct validity

**Fig. 1:**
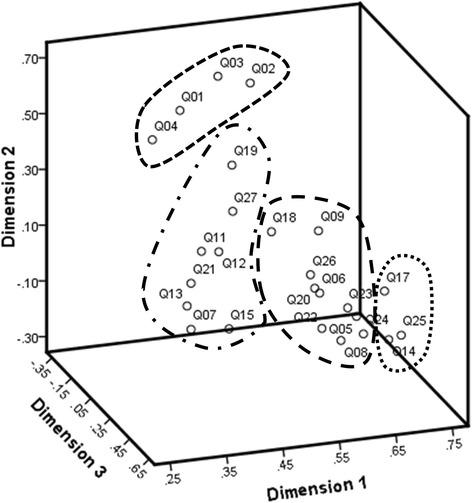
Three-dimensional graph showing the four question subgroups in the Scoliosis Japanese Questionnaire-27

**Fig. 2:**
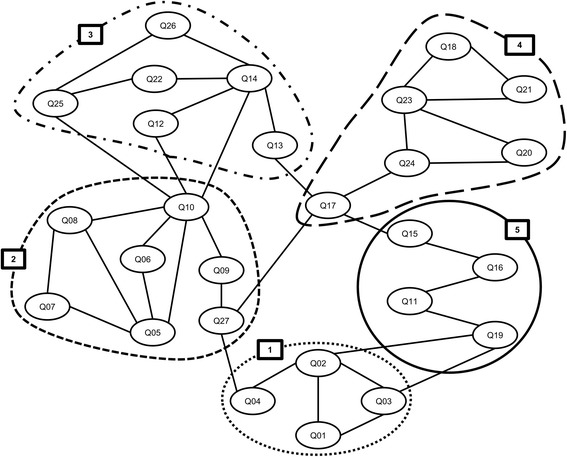
Akaike’s information criterion network for the Scoliosis Japanese Questionnaire-27. Five domains were identified: (1) Pain, (2) Discomfort when wearing clothes, (3) Appearance, (4) Cognition, and (5) Participation

A total of 357 out of 384 participants (93.0%) completed both the SJ-27 and the SRS-22 questionnaires. Spearman’s correlation coefficient between the SJ-27 and the SRS-22 was 0.692 (*p* < 0.001). Four domains were identified using the three-dimensional components plotted with CATPCA (Fig. 1), but these domains were not as clearly categorized as expected; thus, further analysis was required for spatial relationships between these domains, according to the calculated SJ-27 groupings. The AIC calculation of SJ-27 produced 351 (= _27_C_2_) “minimal distance” assortments (degree of independence) for the two-item groupings. Among all the 351 AIC pairs, Q2 and Q3 produced the minimum AIC value (− 252.49), indicating this as the best matched pair. The AIC analysis enabled the division of SJ-27 items into five distinct domains: (1) Pain, (2) Discomfort when wearing clothes, (3) Appearance, (4) Cognition, and (5) Participation, with the five domains being interlinked (Fig. 2). Q17 (“To what extent does your appearance in the mirror make you self-conscious?”) was centered between domains (2) and (4).

## Discussion

A progressive spinal deformity associated with AIS negatively influences a patient’s HRQOL by limiting physical ability and causing back pain, mental disorders, and a negative perception of one’s body [4–6]. A patient’s HRQOL can be determined using patient-reported outcome measures [11] and the SRS scale is currently the gold standard for assessing HRQOL in patients with AIS [16–19, 21]. A Japanese version of the SRS scale has been established, and its reliability and validity were proven [35–38]. However, this scale is thought to be at least partially suboptimal for Japanese patients with AIS because of cultural differences. The need for an original, patient-reported outcome measure for Japanese patients resulted in the development of a new instrument by us, the SJ-27.

The percentage of patients who achieved the lowest score in the SJ-27 and the SRS-22 were 0.5% and 0.0%, respectively. The percentage of patients who achieved the highest score in the SJ-27 and the SRS-22 were 0.0% and 0.3%, respectively. These results indicated that there were no floor and ceiling effects associated with the SJ-27 and the SRS-22 according to the widely used definition of this phenomenon. Based on the results of the current assessment, the SJ-27 showed an excellent internal consistency (Cronbach’s alpha coefficient, 0.914) and a strong correlation with the SRS-22 (Spearman’s correlation efficient, 0.692). With regard to construct validity, the AIC analysis revealed that five distinct domains (Pain, Discomfort when wearing clothes, Appearance, Cognition, and Participation) were identified in the SJ-27 (Fig. 2) and these domains are comparable to the theoretically designed domains originally developed for the SJ-27 questionnaire. These results suggest that the SJ-27 questionnaire covers a wide range of health-related issues, from pain to psychosocial problems. Consequently, our study demonstrates that the SJ-27 questionnaire is a reliable and valid patient-reported outcome measure for female patients with AIS in Japan.

In the formulation of the questions for the SJ-27, we utilized modest and mild expressions, such as that in Q22 (“To what extent do you feel shy or reclusive because of the shape of your spine?”), which reflect the typical modest and reserved characteristics associated with Japanese individuals. These characteristics may also be common in other Eastern countries. Furthermore, we excluded the question on economic burden because of the nature of the Japanese medical insurance system. Accordingly, these considerations may lead to the improved reliability and validity of the SJ-27.

During the development of the SJ-27, we created seven questions regarding scoliosis-related discomfort when wearing clothes or holding bags (Q5–10 and Q27). These items were independently categorized into a distinct domain (“Discomfort when wearing clothes”) using AIC analysis (Fig. 2). Furthermore, Cronbach’s alpha coefficient upon deletion of each item in the SJ-27 (Table 3) revealed that Q10 (“To what extent are you self-conscious about the curve of your back when wearing lighter clothing in warmer weather?”) was a one of the major contributors to its excellent internal consistency. These findings suggest that problems associated with wearing clothes may be closely associated with the HRQOL in patients with AIS and may be important for young female patients who are likely to have a strong interest in fashion. Moreover, the problems associated with wearing clothes in female patients with AIS may arise from changing their dressing habits to conceal their body deformity. Further studies are warranted to determine whether these problems are crucial for female patients with AIS, relative to controls.

An analysis of the relationships between items in the SJ-27 using the AIC method revealed that Q17 (“To what extent does your appearance in the mirror make you self-conscious?”) was centered between domains (2) and (4), indicating that Q17 is a key item for assessing HRQOL in female patients with AIS. These patients usually realize and accept their deformity by observing their body shape in a mirror after being diagnosed with scoliosis or when pointed out by family members or colleagues. We speculate that viewing themselves in mirrors may result in self-image problems among adolescent girls and that these constant visible reminders may negatively influence various aspects of the patient’s HRQOL.

The main purpose of using outcome measures is to examine disease clinical severity, determine the proper timing of therapeutic interventions, and choose the most suitable management method for a patient’s condition. The SRS scale has often been utilized for making decisions regarding the initiation of treatment and evaluating the effects of therapeutic interventions. In the future, we wish to evaluate the relationship between the total SJ-27 score and scoliosis severity (e.g., a Cobb angle >30^o^) and determine the SJ-27 threshold influencing the decision to initiate therapeutic interventions, especially surgery.

This study has several limitations. First, the participants included untreated patients and patients treated with various approaches. However, given that each of the top three treatment methods (brace, observation, and surgery) has little influence on the internal consistency of the SJ-27 (Cronbach’s alpha coefficient of each treatment group: brace, 0.909; observation, 0.903; surgery, 0.916), this instrument may be useful for the assessment of HRQOL in patients with AIS regardless of variation in treatment methods. Second, a healthy control group was not included. Hence, further study is required to validate the SJ-27 by comparing the results of the study group with the results of a control group. Third, some institutions had a small number of female patients with AIS (Table 2), which leads to the possibility of selection bias in this study. Fourth, we did not assess for any physical comorbidities among the participants, which may affect the SJ-27 results. Fifth, we did not specify the timing of the questionnaire administration, whether it should be done prior to or after clinical examination. If patients were given questionnaires after clinical examination, the examination results may have a positive or negative impact on the responses. Finally, test-retest reliability, age effect, and severity effect were not evaluated due to the lack of opportunity to repeatedly administer the SJ-27 to the same participants. In future work, we hope to examine the test-retest reliability, age effect, and severity effect of the SJ-27 by employing longitudinal assessment.

## Conclusions

Our study demonstrated that the SJ-27, which is a novel patient-reported outcome measure for Japanese female patients with AIS, showed good reliability and validity. Therefore, the SJ-27 could assist clinicians in assessing their female patients and help individuals with spinal deformities understand their health status.

## Abbreviations

AIC: Akaike’s information criterion
AIS: Adolescent idiopathic scoliosis
CATDAP: Categorical data analysis program
CATPCA: Categorical principal component analysis
HRQOL: Health-related quality of life
SJ-27: Scoliosis Japanese Questionnaire-27
SRS: Scoliosis Research Society
